# Concept Mapping in Simulation within Nursing Education: A Scoping Review Protocol

**DOI:** 10.3390/nursrep13010011

**Published:** 2023-01-13

**Authors:** Jennifer Innis, Sarah Johnston, Erica Cambly

**Affiliations:** Lawrence S. Bloomberg Faculty of Nursing, University of Toronto, 155 College Street, Toronto, ON M5T 1P8, Canada

**Keywords:** concept mapping, nursing education, simulation, concept formation, scoping review

## Abstract

Background: Simulation has been found to enhance nursing student knowledge and confidence, as well as to improve clinical performance. The use of concept maps during simulation has been found to play a key role in student learning. There is a need to understand what is known to date about the use of concept mapping in simulation within nursing education. This will help determine the most effective ways to use concept mapping in simulation to foster learning in nursing students. Scoping review question: What is known about the context, processes, and outcomes of concept mapping in simulation within nursing education? Methods: The scoping review will be conducted in accordance with JBI methodology for scoping reviews and will search the following databases: MEDLINE, CINAHL, PsycMED, EMBASE, and ERIC. This review will consider studies that explore the use of concept mapping in simulation within undergraduate nursing education and will include studies that have used qualitative, quantitative, and mixed methods, as well as literature reviews. Editorials, commentaries, and gray literature will be excluded. Studies published from 1992 onward will be included. The data extracted will include details about the participants, how concept mapping was used within simulation, methods, key findings, and research gaps.

## 1. Background

Simulation has been used in nursing education for over 100 years, and includes the use of role play, standardized patients, and mannequins. Since the 1990s, high fidelity simulation has been used, which incorporates the use of computerized mannequins, and, more recently, we have seen the growth of virtual simulation. Simulation experiences are designed to resemble and even replicate the clinical environment in which nursing students will practice. Not only does this help nursing students learn new skills, the use of simulation has been found to enhance nursing students’ knowledge and confidence, as well as improve clinical performance. In addition, simulation fosters clinical reasoning and judgement in nursing students [1,2,3].

There is a need to obtain a greater understanding of what teaching and learning strategies can be used to enhance student learning when using simulation [2]. There is a growing understanding that the use of concept maps during simulation plays a key role in student learning. Concept mapping was introduced in the 1980s, and the first research study that examined its use in nursing education was in 1992. Concept mapping involves helping learners to identify concepts that are part of a topic. By identifying general concepts that relate to the topic and naming specific concepts that fall under the general concepts, learners can create links between concepts to develop and synthesize learning [2,3,4].

Concept maps have been found to promote critical thinking and clinical judgement in nursing education. They have also been associated with helping students connect theory and practice by demonstrating how theoretical knowledge can be directly applied to clinical practice with patients and families [2,4,5,6,7,8]. Within simulation, nursing students have reported improvements in critical thinking with the use of concept mapping as a teaching-learning strategy [9].

The use of concept maps in simulation can be used in the pre-briefing phase to support understanding of the learning objectives [10]. This has been found to help nursing students to assess patients during scenarios and to understand complex situations and disease processes [2]. Concept mapping can also be used in debriefing to enhance learner reflection and to assimilate new and pre-existing knowledge and skills [11]. A recent study found that using concept mapping as part of debriefing enhanced students’ critical thinking [7].

There is a need to find out what is known to date about the use of concept mapping in simulation within nursing education. With respect to the use of concept mapping in nursing education, there has been a narrative literature review [4] and a systematic literature review on its impact on the development of critical thinking [8]. Regarding the use of simulation in nursing education, there have been narrative literature reviews [12,13], and a systematic literature review [3]. There is a need to examine the use of concept mapping specifically with respect to simulation. A search of MEDLINE, the Cochrane Database of Systematic Reviews, and the Open Science Framework was performed and found no current or in-progress scoping reviews or systematic reviews on the topic of concept mapping in simulation within nursing education.

The use of concept maps in simulation has been identified as an area in need of further study [2,4]. The use of simulation in nursing education continues to grow with the expanded use of high-fidelity simulation and virtual simulation, especially with the shift to increased online learning with the COVID-19 pandemic. There is a need to obtain an understanding of how concept maps can be used in simulation experiences in nursing education and how they can best foster student learning. The goal of this scoping review is to find out what is known about the context, processes, and outcomes of concept mapping in simulation within nursing education.

## 2. Methods and Analysis

This scoping review will follow the Joanna Briggs Institute (JBI) methodology for scoping reviews [14]. The reporting of this scoping review will be in accordance with the PRISMA Extension for Scoping Reviews (PRISMA-ScR) [15].

The search strategy will aim to locate both published and unpublished primary studies and reviews. A search of MEDLINE (Ovid) and CINAHL (EBSCO) was undertaken to identify articles on the topic. The text words contained in the titles and abstracts of relevant articles and the index terms used to describe the articles were used to develop a full search strategy for MEDLINE (Ovid) and CINAHL (EBSCO) (see Appendix A). The search strategy, including all identified keywords and index terms, will be adapted for the following databases: PsycMED (Ovid), EMBASE (Ovid), and ERIC (ProQuest). The reference lists of articles selected for full text will be screened for additional papers.

Inclusion and exclusion criteria will be used to filter studies written in any language and published between January 1992 and September 2022. The year 1992 was chosen as the start date as this is the first time a nursing study that used concept mapping was published [4].

The participants, concept, and context (PCC) format was used as a guide to develop the search strategy [14,15].

Inclusion criteria:

- Empirical work (qualitative, quantitative, or mixed methods);
- Systematic, scoping, and narrative literature reviews;
- Participants: undergraduate nursing students;
- Concept: concept mapping in simulation;
- Context: academic or clinical setting.
- Exclusion criteria:
- Interventions that do not include concept mapping;
- Types of references: editorials, commentaries, gray literature.

Following the search, all identified records will be collated and uploaded into ENDNOTE version 20 (Clarivate Analytics, Philadelphia, PA, USA) and duplicates removed. Covidence (Veritas Health Innovation, Melbourne, Australia) will be used to manage the screening of titles and abstracts, as well as the full screening of articles. Following a pilot test, titles and abstracts will be screened by two independent reviewers for assessment against the inclusion criteria for the review. Potentially relevant papers will be retrieved in full, and their citation details imported into Covidence (Veritas Health Innovation, Melbourne, Australia). The full text of selected citations will be assessed in detail against the inclusion criteria by two independent reviewers. Reasons for the exclusion of full-text papers that do not meet the inclusion criteria will be noted in the scoping review. Any conflicts between the reviewers during the review process will be resolved through discussion or with a third reviewer. The results of the search will be reported in full in the final scoping review and presented in a PRISMA flow diagram [15].

Data will be extracted from papers included in the scoping review by two independent reviewers with a data extraction tool using Covidence (Veritas Health Innovation, Melbourne, Australia). The data extracted will include specific details about the article that includes author names, the year of the article, participants, how concept mapping was used in the simulation, study setting, country, research methods used, key findings, and research gaps. The data extraction tool will be revised as needed during the process of extracting data from the included articles. Any conflicts that arise between the reviewers will be resolved through discussion or with a third reviewer. If needed, authors of papers will be contacted to request missing or additional information.

The results will be presented in a narrative form. The extracted data will be presented in a table which will summarize information from the articles that is relevant to the goal of this scoping review. The narrative review will describe how the articles address the goal of this scoping review and will identify research gaps.

## 3. Dissemination

The results of this scoping review will be used to identify current practices in the use of concepts maps in simulation within undergraduate nursing education and will identify areas that require more investigation. The results will be disseminated at nursing education meetings and conferences, and via peer-reviewed publication in a nursing journal. The findings of this scoping review will give insight into how concept mapping can best be used as part of simulation within nursing education.

## Data Availability

Not applicable.

## Review Registration Number

Open Science Framework https://osf.io/fd4ya (accessed on 13 September 2022).

## Appendix A. Search Strategy

MEDLINE (Ovid) E: Epub Ahead of Print, In-Process, and Other Non-Indexed Citations, Ovid MEDLINE^®^ Daily and Ovid MEDLINE^®^ <1946-Present>.

Search conducted on 13 September 2022.

**Search**	**Query**	**Records Retrieved**
#1	(concept* adj4 map*).tw,kf. OR (concept* adj4 diagram*).tw,kf OR mind* map*.tw,kf OR know* map*.tw,kf. OR mental map*.tw,kf OR exp Concept Formation/	16,109
#2	exp Simulation Training/ OR simulat*.tw,kf	648,547
#3	exp Students, Nursing/ OR education, nursing, associate/ or education, nursing, baccalaureate/ or education, nursing, diploma programs/ or nursing education research/ OR (student* adj3 nurs*).tw,kf OR (student* adj4 baccalaureate).tw,kf. OR (student* adj4 undergrad*).tw,kf OR (student* adj4 diploma).tw,kf	72,275
#4	#1 AND #2 AND #3	20
Limited to 1992-Current		20

CINAHL Plus with Full Text (EBSCO).

Search conducted on 13 September 2022.

**Search**	**Query**	**Records Retrieved**
#1	MM “Concept Mapping” OR TX concept* N4 map* OR TX mind* map* OR TX know* map* OR TX mental map* OR MM “Concept Formation”	6655
#2	MM “Patient Simulation” OR TX simulat*	122,023
#3	MM “Students, Nursing, Associate” OR MM “Students, Nursing, Baccalaureate” OR MM “Students, Nursing, Diploma Programs” OR MM “Education, Nursing, Research-Based” OR TX nurs* student* OR TX student* N3 nurs* OR TX student* N4 baccalaureate OR TX student* N4 undergrad* OR TX student* N4 diploma	132,219
#4	#1 AND #2 AND #3	433
Limited to 1992-Current		431
